# Supplementary material for: Overcoming data scarcity in biomedical imaging with a foundational multi-task model
Source: Nat Comput Sci. 2024 Jul 19;4(7):495–509. doi: 10.1038/s43588-024-00662-z (PMC11288886; doi:10.1038/s43588-024-00662-z)
Supplement: Supplementary file 1 — Supplementary Sections 1–7, Figs. 1–4 and Tables 1–5. [file 43588_2024_662_MOESM1_ESM.pdf]

nature computational science

Supplementary information

https://doi.org/10.1038/s43588-024-00662-z

Overcoming data scarcity in biomedical imaging with a foundational multi-task model

In the format provided by the authors and unedited

Supplementary Information

Supplementary Section 1 Ablation studies for UMedPT

We performed ablation studies for UMedPT regarding input image size and the choice of normalization layers.

UMedPT-fixed consistently used an image size of 224×224, while UMedPT used the full image dimensions for each task. In our evaluations across various tasks, UMedPT outperformed UMedPT-fixed by 2.97%.

In addition, we tested UMedPT-affine, which also used image dimensions of (224, 224) but added a learnable bias and scaling parameter to UMedPT's static layernorms, adding an affine transformation. Compared to UMedPT-fixed, UMedPT-affine showed an average performance gain of 0.37%. The results are included in Supplementary Tables 3, 4 and 5.

Supplementary Section 2 Benefit of segmentation and object detection in pretraining

To quantify the effect of including multiple label types in the pretraining, we compared UMedPT with a model trained on our classification pretraining tasks only, which we call UMedPT-clf. The results are shown in the Supplementary Figure 2. There is a large average difference and consistently better performance of UMedPT for tasks requiring high spatial resolution features. For the object detection task NucleiDet-WSI, UMedPT achieved a 0.282 higher mean Average Precision (mAP), and for the segmentation task Coloscopy-RGB, it outperformed UMedPT-clf by 0.057 mIoU. Interestingly, although the difference was smaller for Pneumo-CXR (classification), a clear positive knowledge transfer between the label types was found, with an advantage of 2.42% F1-score in favour of UMedPT.

Supplementary Section 3 State of the art for target tasks

Each item in the list corresponds to one reference result in Table 1.

- **CRC-WSI:** Over 94% accuracy in the nine-class classification problem was achieved with ImageNet transfer learning [17].
- **Pneumo-CXR:** The dataset creators reported a diagnostic accuracy of 92.8%, a sensitivity of 93.2%, and a specificity of 90.1% [18].
- **Tuber-CXR:** Radiologists achieved an accuracy of 82% [22]. Specialized representation learning for CXR achieved an AUC of 98.0%, with their corresponding ImageNet baseline reaching an AUC of 94.5% [11].
- **CNS-MRI:** Classifiers with different data splits from the Kaggle community achieved over 96% accuracy.
- **BC-Bach-WSI:** An accuracy of 87% was achieved using external validation on a challenge server [23]
- **BC-BHis-MIC:** The dataset creators reported an accuracy between 80% and 85% [56]. The highest reported F1-score was 88% [24].

Supplementary Table 1 Detailed Performance of UMedPT on MedMNIST. Comparison of accuracy (ACC) and area under the curve (AUC) at different stages of training UMedPT on the MedMNIST database: using the frozen encoder and fine-tuning the whole model. Performance metrics are provided for UMedPT with 1%, 10% and 100% of the training data. Reference results are included from ResNet-50 (Ref. CNN) or the theoretical best results obtained by selecting the method with the strongest test performance for each dataset and metric independently (Ref. Cherrypick). Metrics are reported as mean ± standard deviation in percentage.

	stage	UMedPT - Frozen		UMedPT - Finetune		Ref. Cherrypick		Ref. CNN	
	metric	ACC	AUC	ACC	AUC	ACC	AUC	ACC	AUC
dataset	fraction								
bloodmnist	1%	82.81±1.10%	98.88±0.12%	96.85±0.48%	99.88±0.01%	-	-	-	-
	10%	96.11±0.16%	99.82±0.00%	98.62±0.08%	99.95±0.00%	-	-	-	-
	100%	98.20±0.01%	99.91±0.00%	99.14±0.08%	99.93±0.00%	96.60%	99.80%	95.00%	99.70%
breastmnist	1%	60.68±14.84%	63.07±3.45%	59.62±15.97%	64.89±2.53%	-	-	-	-
	10%	73.72±1.38%	76.95±2.15%	83.76±1.60%	88.28±1.70%	-	-	-	-
	100%	82.91±0.30%	90.99±0.31%	94.02±0.60%	94.41±0.33%	86.30%	91.90%	84.20%	86.60%
chestmnist	1%	94.77±0.03%	72.55±0.60%	94.75±0.05%	74.86±0.88%	-	-	-	-
	10%	94.82±0.01%	78.37±0.15%	94.81±0.01%	80.53±0.09%	-	-	-	-
	100%	94.84±0.00%	80.24±0.01%	94.90±0.01%	84.11±0.04%	94.80%	77.80%	94.80%	77.30%
dermamnist	1%	66.92±0.05%	71.68±2.38%	68.96±0.55%	79.58±2.18%	-	-	-	-
	10%	71.49±0.53%	85.01±1.63%	78.42±0.27%	92.20±0.17%	-	-	-	-
	100%	78.22±0.08%	93.30±0.01%	91.21±0.23%	98.64±0.09%	76.80%	92.00%	73.10%	91.20%
octmnist	1%	69.70±3.31%	97.15±0.08%	84.97±2.19%	98.85±0.14%	-	-	-	-
	10%	76.33±0.61%	97.78±0.09%	88.37±0.12%	98.72±0.24%	-	-	-	-
	100%	77.53±0.39%	97.84±0.02%	92.00±0.83%	99.50±0.16%	77.60%	96.30%	77.60%	95.80%
organamnist	1%	70.95±1.53%	96.52±0.19%	86.69±1.21%	98.93±0.07%	-	-	-	-
	10%	83.28±0.21%	98.49±0.01%	95.16±0.18%	99.80±0.02%	-	-	-	-
	100%	87.01±0.10%	99.04±0.01%	96.86±0.18%	99.89±0.01%	95.10%	99.80%	94.70%	99.80%
organcmnist	1%	38.53±1.45%	88.87±0.73%	70.16±2.19%	95.72±0.45%	-	-	-	-
	10%	72.25±0.27%	96.58±0.04%	89.82±0.36%	99.36±0.05%	-	-	-	-
	100%	81.67±0.12%	98.31±0.00%	95.24±0.13%	99.86±0.01%	92.00%	99.40%	91.10%	99.30%
organsmnist	1%	47.20±3.01%	88.40±0.88%	64.33±0.83%	92.99±0.32%	-	-	-	-
	10%	70.32±0.16%	95.59±0.16%	80.83±1.27%	97.88±0.13%	-	-	-	-
	100%	77.08±0.13%	97.16±0.02%	84.89±0.08%	98.54±0.06%	81.30%	97.50%	78.50%	97.50%
retinamnist	1%	42.50±0.35%	58.07±5.78%	42.42±0.66%	60.64±4.62%	-	-	-	-
	10%	47.08±1.85%	71.69±4.02%	57.75±3.89%	81.63±2.89%	-	-	-	-
	100%	57.00±0.41%	83.96±0.08%	65.83±0.92%	88.57±0.24%	53.10%	75.00%	51.10%	71.60%
tissuemnist	1%	54.86±0.22%	86.12±0.10%	59.52±0.30%	88.80±0.04%	-	-	-	-
	10%	59.32±0.11%	88.74±0.02%	68.06±0.16%	92.78±0.02%	-	-	-	-
	100%	60.21±0.09%	89.28±0.00%	76.63±0.14%	96.06±0.01%	70.30%	94.10%	68.00%	93.20%

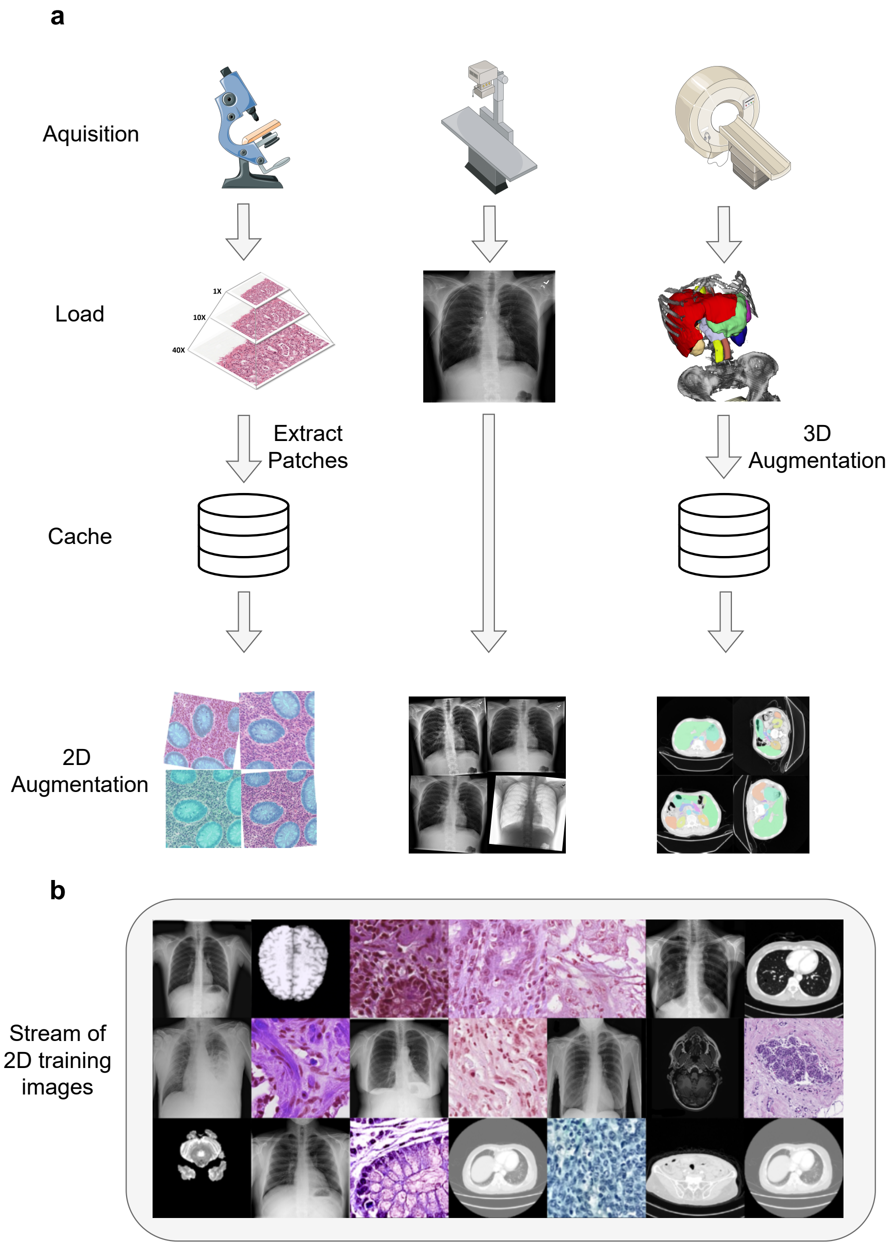

Supplementary Fig. 1 Data pre-processing. **a** We have categorized all of our tasks into one of three common formats: 2D images, 3D tomographic images, and gigapixel images. For each format, we developed a data loading strategy that transforms the data into the required 2D format. Additionally, for each domain, we implemented a standard augmentation strategy to be applied to the corresponding 2D images. **b** Our preprocessing results in a diverse stream of data including samples from all tasks. Image sources: CT [59], CXR [71], WSI [64].

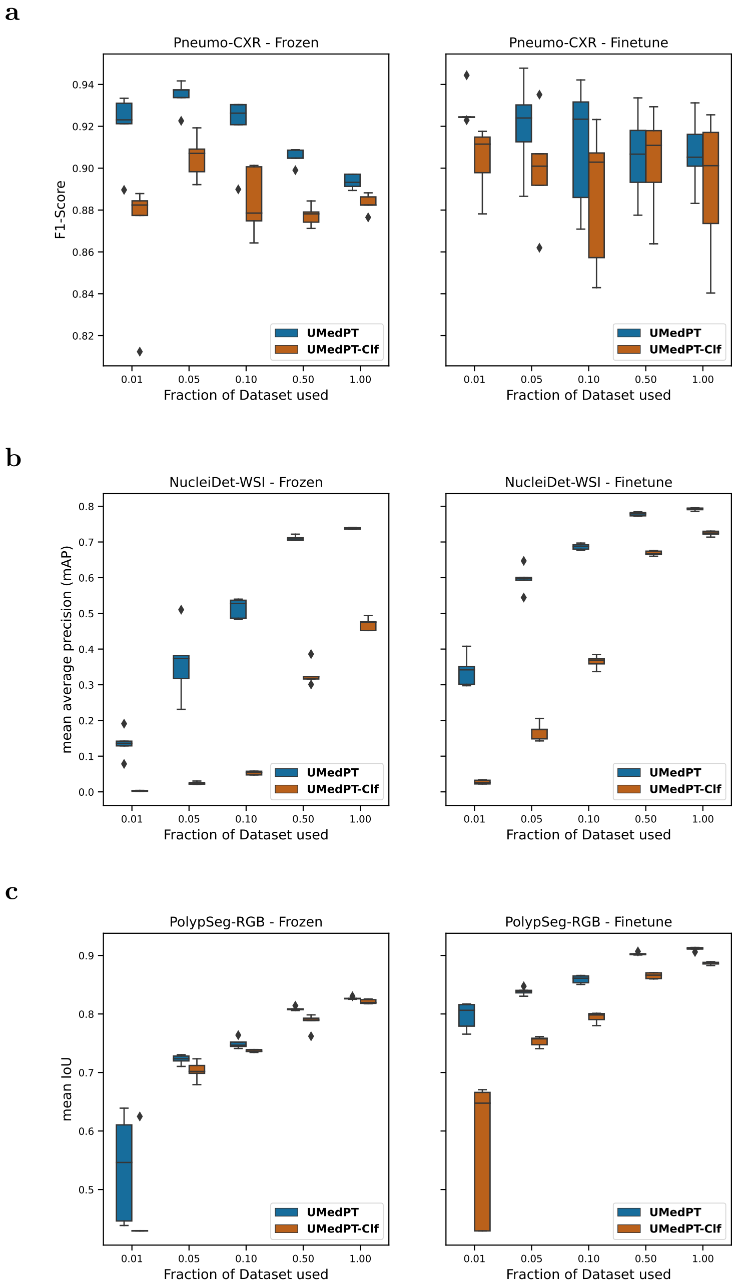

Supplementary Fig. 2 Results of label type ablation study with UMedPT-clf. UMedPT-clf was trained with the same classification tasks as UMedPT, but excluded segmentation and object detection tasks. **a** Pneumo-CXR (classification). **b** NucleiDet-WSI (object detection). **c** PolypSeg-RGB (segmentation). In each setting, 5 independent trainings were derived for each training subset and method. The middle line of the boxes represents the median, the boundaries are the Q1 and Q3 quartiles, the whiskers extend to 1.5 IQR, and outliers beyond are shown as single points.

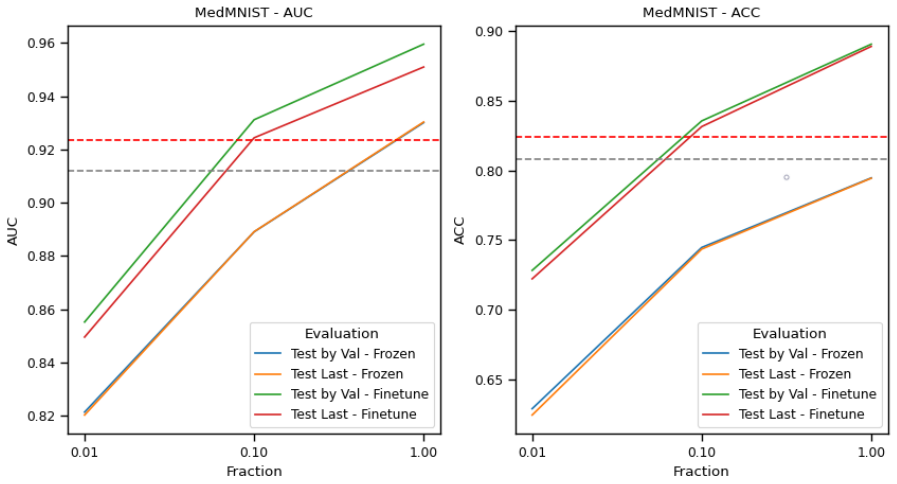

Supplementary Fig. 3 UMedPT's application to MedMNIST. First, UMedPT was applied to MedMNIST [16] with the shared encoder frozen and a randomly initialized linear head (linear probing) and evaluated on the test set using area under the curve (AUC, left) and accuracy (ACC, right). The whole model was then fine-tuned independently for each task. Blue and green lines represent the test performances when the model was selected using the validation set provided by the authors. Red and orange lines represent the test performance when the last model state was selected (validation data not used). Horizontal lines represent the theoretically best performance when the best reference method is selected for each task and metric independently (red) or when the best method is selected for all tasks (grey). We evaluated UMedPT with 1%, 10% and 100% of the training data. Details are given in Supplementary Table 1.

- **PolypSeg-RGB:** The dataset creators [25] reported an mIoU of 0.778. The highest reported mIoU was 0.9051 [26]
- **MedMNIST:** The database creators trained a ResNet-50 on the MedMNIST database and reported an average accuracy of 80.81% and an average AUC of 91.2% [16]. When the best reported method was selected for each metric and task independently ("Ref Cherrypick" in Ext. Data Table 1), the average accuracy was 82.39% and the average AUC was 92.36%.

Supplementary Section 4 Comparing convolutional networks and transformer

For quantifying the effect of the encoder's architecture, we used the MedMNIST database including two-dimensional and three-dimensional classification tasks. We chose the ResNet-50 as convolutional neural network (CNN) and the tiny variant of the Swin Transformer because they are similar in size. The Swin Transformer has 27,582,570 trainable parameters compared to 23,508,032 for the ResNet-50 CNN.

The comparison of the Swin Transformer and ResNet-50 CNN architectures showed a minimal impact on model performance for the MedMNIST database. The Swin Transformer achieved an average test accuracy of 86.76 ± 0.79% over 5 repetitions, while the ResNet-50 CNN achieved an accuracy of 86.34 ±

Supplementary Table 2 Pretraining database statistics. Image instances refer to individual 2D images that can be used directly for pretraining. Composite data types, including 3D volumes and gigapixel images, can be divided into multiple image instances per imaging study. In total, the pretraining database included more than 3 million 2D images, more than 1000 large image tiles such as tissue microarrays or whole slide sections, more than 10000 whole slide images and more than 1000 3D volumes, totalling more than 10 million annotated image instances for pretraining UMedPT. For the dataset splits provided by the respective publishers, we marked them as train/test and included only the training set in the pretraining.

Identifer	Description	Dataset size
Amos22-CT	Segmentation of 15 organs in abdominal CT.	200 3D-CT volumes
Conic-WSI	Nuclei detection in colon tissue from 6 different data sources with 6 classes.	4981 image instances
PICAL-MRI	Multilabel classification of clinically relevant prostate cancer (csPCa) and whether or not a lesion is visible.	1476 cases, each with 3 3D-MR sequences
Panda-WSI-Clf<br>Panda-WSI-Seg	This data source yields two pretraining tasks. A classification task for predicting presence of tumor, and a segmentation task with the classes stroma, healthy epithelium and gleason grades 3, 4 and 5.	10616 WSI
VinBigData-CXR	Object detection in chest X-ray with 14 classes.	15000 chest X-rays
Crag-WSI	Colorectal adenocarcinoma gland segmentation.	213 image tiles (size $\approx (1500, 1500)$
Brats2020-MRI	Brain tumor (glioma) segmentation into five classes.	369 cases. Each case comes with 4 3D-MR sequences.
CRC-WSI	Multi-class classification of H&E stained histological images of human colorectal cancer (CRC).	100,000/7,000 image instances extracted from 86 WSI
Avaniti-WSI	Multi-label classification into four classes (benign and 3 gleason grades). We extracted patches from tissue microarrays and predict all classes present.	886 TMA
Cyto-WSI	Expert-labelled single-cell images taken from peripheral blood smears. Used as a multi-class classification task with 21 classes.	137076 image instances
Chexpert-CXR	Multilabel classification in chest X-ray with 14 classes. We use the nine classes that have a good performance when measured with the provided validation set.	223414/234 image instances
SIIM-CXR	Segment the pneumothorax area in chest X-ray.	11583 image instances
ImageNet	Multi-class natural image classification dataset with 1000 classes.	1,281,167/50,000 images
RadImageNet	Multi-class classification database developed for the purpose of pretraining medical AI. Contains 2D image instances from CT, MR and ultrasound (US)	263118/29235 CT<br>605408/67267 MR<br>350897/38988 US
COCO-Seg<br>COCO-Det	Natural image dataset with 80 segmentation and object detection classes.	118287 image instances

1.01%. In addition, a discrepancy in training convergence rates was observed between the two architectures, as shown in Extended Data Fig. 2a.

Supplementary Section 5 Investigating training schemes

We performed an analysis of training schemes on the MedMNIST database, including the two- and three-dimensional tasks. All datasets in MedMNIST

Supplementary Table 3 In-domain benchmark results. The left pair of columns shows results with a frozen encoder, while the right pair shows results with fine-tuning. F1-scores are reported as mean ± standard deviation in percentage. P-values, calculated independently by a paired one-sided t-test for each dataset size, were always compared against the pretrained ImageNet-1K model. UMedPT-fixed was an ablation study where patch sizes remained constant, and UMedPT-affine was another ablation where layernorms had trainable parameters. Unless otherwise stated, all results in the main paper were obtained with UMedPT.

		frozen		finetune	
Size	Model	Pneumo-CXR	CRC-WSI	Pneumo-CXR	CRC-WSI
1%	ImageNet	61.68±12.55%	80.81±0.54%	85.54±2.32%	91.84±1.70%
	UMedPT fixed	89.06±1.39% p=4.54e-03	97.03±0.07% p=3.13e-07	91.52±1.97% p=2.19e-02	96.53±0.61% p=2.58e-03
	UMedPT affine	88.51±1.71% p=5.54e-03	97.17±0.08% p=2.96e-07	91.71±1.97% p=1.74e-02	96.98±0.76% p=7.02e-04
	UMedPT	91.97±1.57% p=3.09e-03	95.37±0.13% p=8.26e-07	92.81±0.82% p=2.18e-03	95.97±0.64% p=2.32e-03
5%	ImageNet	78.07±1.27%	84.91±0.35%	84.80±1.99%	94.64±0.82%
	UMedPT fixed	89.49±1.29% p=8.35e-05	96.96±0.11% p=2.27e-07	91.34±1.84% p=1.79e-03	96.57±0.34% p=3.98e-03
	UMedPT affine	88.24±1.44% p=9.74e-05	96.96±0.15% p=1.82e-08	91.04±2.01% p=5.33e-04	96.78±0.30% p=7.52e-03
	UMedPT	93.46±0.65% p=1.14e-06	95.45±0.14% p=6.89e-07	92.02±2.03% p=6.77e-04	95.68±0.70% p=7.90e-02
10%	ImageNet	79.05±1.54%	86.13±0.35%	85.06±6.84%	95.44±0.56%
	UMedPT fixed	87.63±2.51% p=1.02e-04	97.04±0.17% p=2.65e-07	90.05±3.06% p=8.43e-02	96.45±0.32% p=5.16e-03
	UMedPT affine	87.50±2.86% p=2.06e-04	96.91±0.22% p=1.99e-07	89.79±2.62% p=8.19e-02	95.83±0.46% p=1.25e-01
	UMedPT	91.95±1.52% p=2.16e-05	95.20±0.40% p=7.47e-06	91.08±2.75% p=3.12e-02	95.55±0.61% p=3.93e-01
50%	ImageNet	81.53±0.57%	87.61±0.12%	88.81±2.85%	95.69±0.68%
	UMedPT fixed	89.56±0.76% p=3.93e-05	96.83±0.13% p=1.72e-08	90.50±3.02% p=1.91e-02	95.70±0.68% p=4.90e-01
	UMedPT affine	89.65±0.64% p=2.60e-05	96.70±0.12% p=4.67e-11	92.16±1.04% p=3.42e-02	95.78±0.76% p=4.37e-01
	UMedPT	90.52±0.35% p=2.26e-07	95.59±0.13% p=4.43e-08	90.58±1.94% p=2.22e-01	95.01±0.69% p=8.68e-01
100%	ImageNet	82.18±0.94%	87.68±0.07%	90.34±1.77%	95.16±0.59%
	UMedPT fixed	90.14±0.66% p=8.95e-06	96.82±0.08% p=4.69e-10	90.17±0.44% p=5.83e-01	94.93±0.61% p=6.80e-01
	UMedPT affine	90.01±0.48% p=2.18e-05	96.59±0.02% p=4.60e-10	90.61±2.28% p=4.42e-01	95.60±0.33% p=9.77e-02
	UMedPT	89.36±0.31% p=3.40e-05	95.57±0.06% p=1.43e-09	90.73±1.60% p=3.65e-01	94.98±1.09% p=5.94e-01

Supplementary Table 4 Out-of-domain benchmark with frozen encoder. All tasks were analysed using F1-score. Metrics are reported as mean ± standard deviation in percentage. P-values, calculated independently by a paired one-sided t-test for each dataset size, were always compared against the pretrained ImageNet-1K model. UMedPT-fixed was an ablation study where patch sizes remained constant, and UMedPT-affine was another ablation where layernorms had trainable parameters.

Size	Model	Tuber-CXR	CNS-MRI	BC-BHis-MIC	BC-BACH-WSI
1%	ImageNet	37.88±5.86%	31.49±9.61%	47.94±7.05%	32.98±3.58%
	UMedPT fixed	55.70±14.94% p=2.27e-02	31.69±9.12% p=4.73e-01	47.79±7.68% p=5.20e-01	26.99±5.51% p=8.87e-01
	UMedPT affine	55.66±13.27% p=1.75e-02	37.18±7.00% p=7.53e-02	55.14±11.11% p=6.43e-02	33.19±8.92% p=4.82e-01
	UMedPT	51.63±16.87% p=4.38e-02	69.18±5.68% p=7.19e-05	46.94±5.56% p=6.24e-01	34.64±10.00% p=3.94e-01
5%	ImageNet	46.87±14.45%	75.14±2.31%	45.41±3.91%	20.19±10.31%
	UMedPT fixed	67.62±3.28% p=1.11e-02	80.21±2.85% p=1.06e-03	55.47±4.68% p=4.58e-03	26.26±9.31% p=1.64e-01
	UMedPT affine	68.30±6.40% p=2.77e-02	78.15±3.69% p=7.35e-03	61.37±9.69% p=4.71e-03	33.10±13.31% p=6.64e-02
	UMedPT	76.67±16.65% p=5.88e-03	86.80±0.76% p=1.35e-04	74.23±2.77% p=6.52e-06	29.78±12.12% p=8.18e-02
10%	ImageNet	50.42±11.04%	80.03±0.84%	57.23±13.31%	39.90±8.90%
	UMedPT fixed	76.18±2.57% p=1.97e-03	84.82±0.74% p=8.57e-06	61.34±11.47% p=2.13e-01	47.70±15.34% p=7.10e-02
	UMedPT affine	76.25±4.73% p=1.87e-03	84.22±1.04% p=7.93e-05	68.03±8.56% p=1.86e-02	51.29±14.69% p=5.00e-02
	UMedPT	86.42±2.12% p=9.65e-04	88.95±0.33% p=5.82e-06	79.17±2.12% p=9.91e-03	56.83±14.76% p=4.62e-03
50%	ImageNet	70.42±4.14%	87.83±0.81%	77.76±0.93%	61.24±2.71%
	UMedPT fixed	85.03±1.10% p=1.56e-03	91.65±0.28% p=5.36e-04	84.12±0.26% p=2.45e-05	70.63±6.97% p=2.59e-02
	UMedPT affine	84.18±1.32% p=1.78e-03	90.68±0.38% p=3.27e-03	85.18±1.42% p=1.37e-03	75.92±3.07% p=2.17e-04
	UMedPT	90.50±0.91% p=3.88e-04	92.95±0.33% p=1.53e-04	86.94±1.14% p=4.44e-05	77.97±0.96% p=1.84e-04
100%	ImageNet	67.99±1.11%	89.05±0.11%	82.30±0.58%	72.90±1.49%
	UMedPT fixed	86.89±0.93% p=6.22e-07	93.33±0.11% p=3.37e-07	87.15±0.31% p=1.32e-04	80.98±1.28% p=1.09e-03
	UMedPT affine	86.01±0.25% p=1.76e-06	92.82±0.20% p=1.19e-06	87.12±0.22% p=4.94e-05	85.84±1.24% p=1.87e-04
	UMedPT	93.50±0.20% p=1.12e-06	94.14±0.17% p=1.60e-06	89.87±0.22% p=1.11e-05	81.17±0.43% p=2.39e-04

Supplementary Table 5 Out-of-domain benchmark with fine-tuned encoder. All tasks were analysed using F1-score. Metrics are reported as mean ± standard deviation in percentage. P-values, calculated independently by a paired one-sided t-test for each dataset size, were always compared against the pretrained ImageNet-1K model. UMedPT-fixed was an ablation study where patch sizes remained constant, and UMedPT-affine was another ablation where layernorms had trainable parameters.

Size	Model	Tuber-CXR	CNS-MRI	BC-BHis-MIC	BC-BACH-WSI
1%	ImageNet	58.21±9.50%	69.24±2.81%	68.96±6.90%	36.44±5.12%
	UMedPT fixed	62.70±8.92%<br>p=2.98e-01	79.46±1.60%<br>p=3.81e-03	70.87±7.19%<br>p=1.29e-01	38.07±7.64%<br>p=3.29e-01
	UMedPT affine	61.47±11.45%<br>p=3.53e-01	75.81±6.93%<br>p=2.08e-02	69.72±6.94%<br>p=3.71e-01	34.81±5.74%<br>p=6.75e-01
	UMedPT	69.54±12.54%<br>p=1.11e-01	84.77±2.83%<br>p=4.98e-04	68.79±8.92%<br>p=5.29e-01	41.19±6.57%<br>p=1.34e-01
5%	ImageNet	77.05±3.36%	91.21±0.78%	86.77±2.49%	47.62±7.25%
	UMedPT fixed	83.44±1.85%<br>p=9.78e-03	93.57±0.36%<br>p=8.46e-04	88.45±1.63%<br>p=1.22e-02	43.02±6.84%<br>p=9.43e-01
	UMedPT affine	82.44±2.88%<br>p=2.55e-02	93.01±0.80%<br>p=7.60e-04	86.39±1.30%<br>p=6.79e-01	42.42±8.40%<br>p=9.44e-01
	UMedPT	92.51±2.36%<br>p=2.80e-04	93.50±0.74%<br>p=6.54e-03	87.41±1.96%<br>p=1.27e-01	47.61±12.52%<br>p=5.01e-01
10%	ImageNet	81.17±2.49%	93.53±1.43%	89.29±1.55%	59.84±8.95%
	UMedPT fixed	89.64±0.98%<br>p=1.38e-03	95.27±0.66%<br>p=1.12e-02	91.86±0.53%<br>p=1.07e-02	66.42±8.97%<br>p=6.17e-03
	UMedPT affine	88.34±2.59%<br>p=9.94e-03	95.24±0.65%<br>p=7.05e-03	91.15±0.57%<br>p=6.92e-02	64.51±9.56%<br>p=6.65e-03
	UMedPT	96.25±1.75%<br>p=5.86e-04	95.62±0.47%<br>p=7.18e-03	91.60±0.72%<br>p=3.11e-02	66.54±11.62%<br>p=7.55e-03
50%	ImageNet	88.80±2.20%	98.33±0.19%	95.97±1.44%	83.55±5.42%
	UMedPT fixed	93.01±0.66%<br>p=3.84e-03	98.30±0.16%<br>p=5.78e-01	97.21±0.49%<br>p=3.57e-02	89.58±3.20%<br>p=2.23e-02
	UMedPT affine	92.61±0.24%<br>p=1.20e-02	98.24±0.15%<br>p=6.93e-01	96.65±0.85%<br>p=5.07e-02	87.87±3.09%<br>p=9.95e-02
	UMedPT	95.44±0.72%<br>p=7.40e-04	98.63±0.11%<br>p=2.66e-02	97.20±0.80%<br>p=1.43e-02	88.81±2.79%<br>p=5.68e-02
100%	ImageNet	90.13±4.98%	98.98±0.13%	98.39±0.35%	92.99±2.24%
	UMedPT fixed	94.13±0.82%<br>p=9.53e-02	99.27±0.06%<br>p=1.25e-02	98.83±0.19%<br>p=5.34e-02	95.54±1.33%<br>p=6.15e-02
	UMedPT affine	93.12±0.52%<br>p=1.48e-01	99.15±0.20%<br>p=6.26e-02	98.23±0.45%<br>p=6.47e-01	94.54±1.61%<br>p=2.09e-01
	UMedPT	93.92±0.46%<br>p=1.11e-01	99.27±0.15%<br>p=1.65e-02	98.38±0.60%<br>p=5.07e-01	92.67±2.10%<br>p=5.64e-01

have a fixed number of cases. This distinction enabled us to conduct ablation studies comparing infinite task sampling with balanced sampling based on dataset size. Besides this, we used the same training schedule and hyperparameters as in the main study, and accumulated the gradients of as many steps as there were tasks. In addition, for comparison with traditional training schemes, we used the same setting without gradient accumulation and also with the SGD optimizer instead of Adam.

The exploration of training schemes showed that balanced (by dataset size) and cyclic sampling (as in UMedPT) exhibited similar behaviour in terms of convergence. However, balanced sampling occasionally showed reduced stability; it yielded a standard deviation of 1.81±1.79% in validation accuracy over the previous ten epochs, across five different experiments. In comparison, cyclic sampling showed a more stable training process, achieving a comparatively lower standard deviation of 1.17±1.09%. When gradient accumulation was excluded, the resulting performance deteriorated, accompanied by longer convergence times. These results are shown in Extended Data Fig. 2b.

Supplementary Section 6 Inverse relationship between performance and dataset size

Our evaluation within the clinical benchmark revealed an unexpected trend in some datasets: increasing the dataset size for fine-tuning sometimes led to a decrease in model performance.

To investigate the potential influence of *catastrophic forgetting* [35] or overfitting during fine-tuning, we first evaluated this phenomenon using four MedMNIST tasks that had shown improved performance with multi-task learning compared to single-task learning. We first measured the test accuracy of these tasks after multi-task learning, followed by further individualised training with the full dataset of each task, and assessed the test accuracy again. The results varied between datasets, suggesting that whether datasets are affected by forgetting the well generalizing state from multi-task learning is inconsistent and may be task-dependent:

- SynapseMNIST3D: 83.81±0.31% → 82.90±0.66% (decrease)
- VesselMNIST3D: 93.66±0.31% → 93.77±0.87% (no decrease)
- BreastMNIST: 86.92±1.04% → 85.90±0.91% (decrease)
- PneumoniaMNIST: 91.54±0.48% → 91.70±0.49% (no decrease)

For our in-domain and out-of-domain target tasks, we always used 100 epochs. Consequently, larger datasets used more optimization steps and could overfit more easily. We investigated by keeping large validation sets (30% of the full training data) in one in-domain and one out-of-domain task where the phenomenon occurred and performed model selection using the validation set. Supplementary Figure 4 shows that for one task the model selection with the validation set was better, for the other task it was worse.

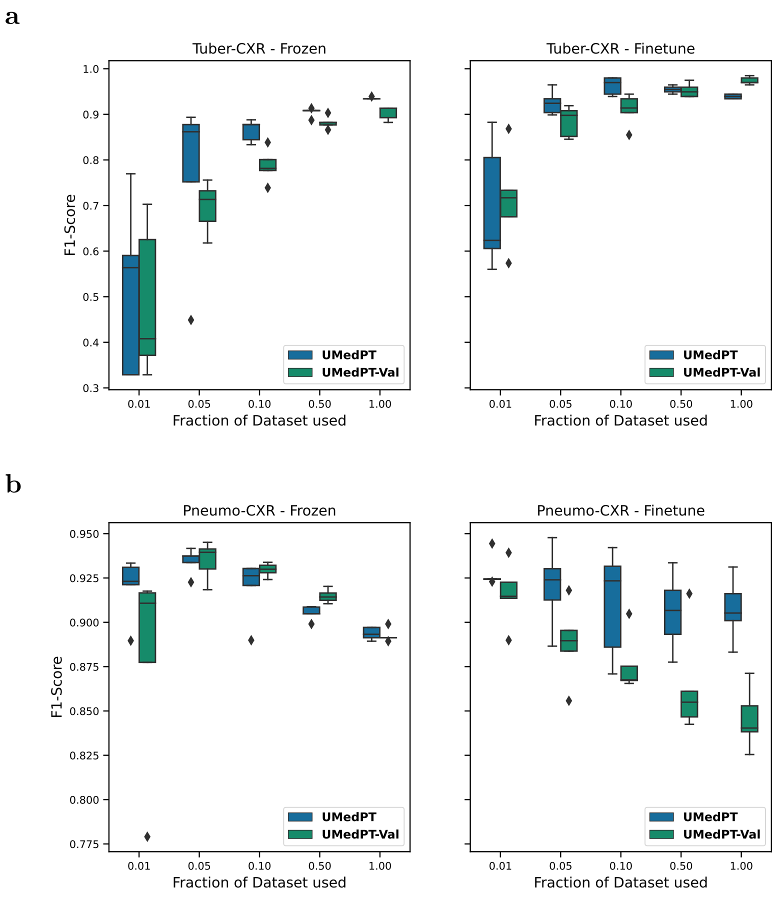

Supplementary Fig. 4 Model selection with and without validation sets. For the target tasks in our clinical benchmark, we did not use validation sets to really only use the given percentage of training data (UMedPT). This could lead to overfitting on the training data, which is usually solved by using a validation set, as done with UMedPT-Val. We investigated this using a representative out-of-domain data set, Tuber-CXR (a), and an in-domain target task, Pneumo-CXR (b). In each setting, 5 independent trainings were derived for each training subset and method. The middle line of the boxes represents the median, the boundaries are the Q1 and Q3 quartiles, the whiskers extend to 1.5 IQR, and outliers beyond are shown as single points.

Supplementary Section 7 Investigating the Applicability to 3D Segmentation Tasks

To evaluate the application of a stacked 2D segmentation approach to 3D images, we examined a lung nodule segmentation task from the medical segmentation decathlon [28]. For compatibility with the benchmark's results, we retrained UMedPT with the nine remaining tasks from the decathlon's dataset

in addition to UMedPT's training database. We refer to this version of the model as UMedPT-large.

The pretraining methodology for UMedPT-large was the same as for UMedPT. To adapt to the target task, we then trained a linear task-specific head on the output of the frozen UMedPT-large. The model was trained using full slices. For inference, we used 2D inference on full slices and stacked the results to create a 3D prediction.

We compared with nnU-Net [51], as a baseline for medical 3D segmentation. While we used whole slices (512 $\times$ 512) for training, nnU-Net used a patch size of 128 $\times$ 128 $\times$ 128. Our evaluation strategy followed the baseline's approach of 5-fold cross validation. For evaluation, we adopted the 3D Dice from [50], reporting only the foreground class. In terms of results, UMedPT-large achieved a Dice score of 71.96%, while for non-pretrained nnU-Net 52.68% and 66.85% are reported for 2D and 3D, respectively [51]. However, at the time of writing, the online leaderboard of the Medical Segmentation Decathlon reports higher metrics (using different test data).

For future work, we suggest following the workflow that was successful with the external evaluation of a colorectal cancer classifier in gigapixel image classification. In this process, we first used UMedPT to extract features, followed by the application of a smaller specialized CNN to the whole gigapixel image at once. For 3D segmentation, this specialized network could be a 3D CNN. Alternatively, the pretraining segmentation task could be extended to incorporate 3D spatial context as we did for 3D classification with MedMNIST.

Supplementary Section 8 List of data sources

Below is a list of the data sources used in this study. All data are either publicly available or can be obtained by requesting access from the respective authors at the URLs listed.

- Amos22 [59] (organ segmentation in CT): https://amos22.grand-challenge.org/
- Conic-WSI [60] (cell detection): https://conic-challenge.grand-challenge.org/
- PICAL-MRI [61] (prostate cancer classification) https://pi-cai.grand-challenge.org/:
- Panda-WSI [62] (prostate tissue semantic segmentation & classification): https://www.kaggle.com/c/prostate-cancer-grade-assessment
- VinBigData-CXR [63] (Thorax pathology pathology detection): https://www.kaggle.com/competitions/vinbigdata-chest-xray-abnormalities-detection
- Crag-WSI [64] (Colorectal tissue semantic segmentation): https://github.com/XiaoyuZHK/CRAG-Dataset_Aug_ToCOCO
- Brats2020-MRI [65–67] (brain semantic segmentation): https://www.kaggle.com/datasets/awsaf49/brats20-dataset-training-validation

- Avaniti-WSI [68] (prostate multi-label classification): https://doi.org/10.7910/DVN/OCYCMP
- Cyto-WSI [69] (bone marrow single cell multi-class classification): https://wiki.cancerimagingarchive.net/pages/viewpage.action?pageId=101941770
- Chexpert-CXR [70] (Thorax pathology multi-label classification): https://stanfordaimi.azurewebsites.net/datasets/8cbd9ed4-2eb9-4565-affc-111cf4f7ebe2 & https://github.com/rajpurkarlab/cheXpert-test-set-labels
- SIIM-CXR [71] (pneumothorax semantic segmentation): https://www.kaggle.com/competitions/siim-acr-pneumothorax-segmentation/data
- ImageNet-1K [1] (real world image classification): https://www.image-net.org/download.php
- RadImageNet [5] (radiology multi-class classification): request access at https://www.radimagenet.com/copy-of-home-1
- COCO [72] (real world semantic segmentation & object detection): https://cocodataset.org/#download
- CRC-WSI [17] (colorectal cancer tissue classification): https://zenodo.org/record/1214456
- Pneumo-CXR [18] (pneumonia in pediatric cohort): https://data.mendeley.com/datasets/rscbjbr9sj/3
- Tuber-CXR [20] (tuberculosis diagnosis in CXR): https://www.kaggle.com/datasets/raddar/tuberculosis-chest-xrays-shenzhen
- CNS-MRI [21] (CNS neoplasia diagnosis in MRI): https://www.kaggle.com/datasets/masoudnickparvar/brain-tumor-mri-dataset
- BC-Bach-WSI [23] (breast cancer classification in WSI): https://iciar2018-challenge.grand-challenge.org/
- BC-BHis-MIC [56] (breast cancer classification in microscopic images): https://web.inf.ufpr.br/vri/databases/breast-cancer-histopathological-database-breakhis/
- PolypSeg-RGB [25] (polyp segmentation in coloscopy): https://datasets.simula.no/kvasir-seg/
- NucleiDet-WSI [19] (detection of nuclei in whole slide images): https://www.nature.com/articles/s41597-020-0528-1
- Medical Segmentation Decathlon [28] (3D segmentation experiment): https://decathlon-10.grand-challenge.org/
- MedMNIST database [16] (Application of UMedPT to MedMNIST and separate experiments with MedMNIST): https://zenodo.org/records/5208230
