# Supplementary material for: Overcoming data scarcity in biomedical imaging with a foundational multi-task model
Source: Nat Comput Sci. 2024 Jul 19;4(7):495–509. doi: 10.1038/s43588-024-00662-z (PMC11288886; doi:10.1038/s43588-024-00662-z)
Supplement: Supplementary file 2 — Reporting Summary [file 43588_2024_662_MOESM2_ESM.pdf]

Corresponding author(s): Fabian Kiessling, Johannes Lotz

Last updated by author(s): May 29, 2024

Reporting Summary

Nature Portfolio wishes to improve the reproducibility of the work that we publish. This form provides structure for consistency and transparency in reporting. For further information on Nature Portfolio policies, see our Editorial Policies and the Editorial Policy Checklist.

Statistics

For all statistical analyses, confirm that the following items are present in the figure legend, table legend, main text, or Methods section.

n/a	Confirmed	
☐	☒	The exact sample size (*n*) for each experimental group/condition, given as a discrete number and unit of measurement
☐	☒	A statement on whether measurements were taken from distinct samples or whether the same sample was measured repeatedly
☐	☒	The statistical test(s) used AND whether they are one- or two-sided<br>*Only common tests should be described solely by name; describe more complex techniques in the Methods section.*
☐	☒	A description of all covariates tested
☐	☒	A description of any assumptions or corrections, such as tests of normality and adjustment for multiple comparisons
☐	☒	A full description of the statistical parameters including central tendency (e.g. means) or other basic estimates (e.g. regression coefficient) AND variation (e.g. standard deviation) or associated estimates of uncertainty (e.g. confidence intervals)
☐	☒	For null hypothesis testing, the test statistic (e.g. F, t, r) with confidence intervals, effect sizes, degrees of freedom and P value noted<br>*Give P values as exact values whenever suitable.*
☒	☐	For Bayesian analysis, information on the choice of priors and Markov chain Monte Carlo settings
☐	☒	For hierarchical and complex designs, identification of the appropriate level for tests and full reporting of outcomes
☒	☐	Estimates of effect sizes (e.g. Cohen's *d*, Pearson's *r*), indicating how they were calculated

Our web collection on statistics for biologists contains articles on many of the points above.

Software and code

Policy information about availability of computer code

Data collection	We provide a full list of software used and their exact versions within the code archive. In particular, we used Python (3.10.6) & PyTorch for training deep neural networks. MONAI (1.1.0) and Albumentations (1.3.0) for data preprocessing. We have included an exact list of a installed dependencies in the code archives for each version available from Zenodo (https://zenodo.org/records/11383543): code/medmnist_version/requirements.txt and code/original_version/requirements.txt. The latest version of our training framework is available at https://github.com/FraunhoferMEVIS/MedicalMultitaskModeling, and the latest version of UMedPT is available at https://github.com/FraunhoferMEVIS/UMedPT.
Data analysis	- Pandas (1.5.3)<br>- Scipy (1.10.1)<br>- Matplotlib (3.8.2)<br>- Seaborn (0.13.1)<br>All dependencies are available from the public Python Package Index.

For manuscripts utilizing custom algorithms or software that are central to the research but not yet described in published literature, software must be made available to editors and reviewers. We strongly encourage code deposition in a community repository (e.g. GitHub). See the Nature Portfolio guidelines for submitting code & software for further information.

Data

Policy information about availability of data

All manuscripts must include a data availability statement. This statement should provide the following information, where applicable:

- Accession codes, unique identifiers, or web links for publicly available datasets
- A description of any restrictions on data availability
- For clinical datasets or third party data, please ensure that the statement adheres to our policy

Source Data for Figures 1, 3, 4 and 5 are available with this manuscript. The training and evaluation data were obtained from their original repositories and selected based on availability, clinical relevance, and satisfactory performance metrics.All data sources are listed below:

- Amos22 (organ segmentation in CT): https://amos22.grand-challenge.org/
- Conic-WSI (cell detection): https://conic-challenge.grand-challenge.org/
- PICAL-MRI (prostate cancer classification) https://pi-cai.grand-challenge.org/:
- Panda-WSI (prostate tissue semantic segmentation \& classification): https://www.kaggle.com/c/prostate-cancer-grade-assessment
- VinBigData-CXR hest-xray-abnormalities-detection (Thorax pathology pathology detection): https://www.kaggle.com/competitions/vinbigdata-chest-xray-abnormalities-detection
- Crag-WSI (Colorectal tissue semantic segmentation): https://github.com/XiaoyuZHK/CRAG-Dataset_Aug_ToCOCO
- Brats2020-MRI (brain semantic segmentation): https://www.kaggle.com/datasets/awsaf49/brats20-dataset-training-validation
- Avaniti-WSI (prostate multi-label classification): https://doi.org/10.7910/DVN/OCYCMP
- Cyto-WSI (bone marrow single cell multi-class classification): https://wiki.cancerimagingarchive.net/pages/viewpage.action?pageId=101941770
- Chexpert-CXR (Thorax pathology multi-label classification): https://stanfordaimi.azurewebsites.net/datasets/8cbd9ed4-2eb9-4565-affc-111cf4f7ebe2 \& https://github.com/rajpurkarlab/cheXpert-test-set-labels
- SIIM-CXR (pneumothorax semantic segmentation): https://www.kaggle.com/competitions/siim-acr-pneumothorax-segmentation/data
- ImageNet (real world image classification): https://www.image-net.org/download.php
- RadImageNet (radiology multi-class classification): request access at https://www.radimagenet.com/copy-of-home-1
- COCO (real world semantic segmentation \& object detection): https://cocodataset.org/#download
- CRC-WSI (colorectal cancer tissue classification): https://zenodo.org/record/1214456
- Pneumo-CXR (pneumonia in pediatric cohort): https://data.mendeley.com/datasets/rscbjbr9sj/3
- Tuber-CXR (tuberculosis diagnosis in CXR): https://www.kaggle.com/datasets/raddar/tuberculosis-chest-xrays-shenzhen
- CNS-MRI (CNS neoplasia diagnosis in MRI): https://www.kaggle.com/datasets/masoudnickparvar/brain-tumor-mri-dataset
- BC-Bach-WSI (breast cancer classification in WSI): https://iciar2018-challenge.grand-challenge.org/
- BC-BHis-MIC (breast cancer classification in microscopic images): https://web.inf.ufpr.br/vri/databases/breast-cancer-histopathological-database-breakhis/
- PolypSeg-RGB (polyp segmentation in coloscopy): https://datasets.simula.no/kvasir-seg/
- NucleiDet-WSI (detection of nuclei in whole slide images): https://www.nature.com/articles/s41597-020-0528-1
- Medical Segmentation Decathlon (3D segmentation experiment): https://decathlon-10.grand-challenge.org/
- MedMNIST database (Application of UMedPT to MedMNIST and separate experiments with MedMNIST): https://zenodo.org/records/5208230

All data are either directly publicly accessible or can be obtained by requesting access at the specified URL from the authors of the dataset.

Research involving human participants, their data, or biological material

Policy information about studies with human participants or human data. See also policy information about sex, gender (identity/presentation), and sexual orientation and race, ethnicity and racism.

Reporting on sex and gender	Not applicable. For reproducibility, we use all cohorts as provided by their publishers.
Reporting on race, ethnicity, or other socially relevant groupings	Not applicable. For reproducibility, we use all cohorts as provided by their publishers.
Population characteristics	Not applicable.
Recruitment	Not applicable.
Ethics oversight	Not applicable.

Note that full information on the approval of the study protocol must also be provided in the manuscript.

Field-specific reporting

Please select the one below that is the best fit for your research. If you are not sure, read the appropriate sections before making your selection.

☒ Life sciences ☐ Behavioural & social sciences ☐ Ecological, evolutionary & environmental sciences

For a reference copy of the document with all sections, see nature.com/documents/nr-reporting-summary-flat.pdf

Life sciences study design

All studies must disclose on these points even when the disclosure is negative.

Sample size	We used 23 independent, external data sets that are publicly available. In consequence, the sample size for all data sources was predetermined. However, for simulating data scarcity we chose random subsets of the full datasets at 1%, 5%, 10%, 50% and 100% by intuition. For experiments with MedMNIST, we chose 1%, 10%, and 100% to simulate data scarcity due to the large number of experiments. Given the available computational resources, we chose the number of subsamples and the number of replicates per experimental setting (n=5 each) to be as large as possible.
Data exclusions	No data exclusions.
Replication	We make all code publicly available and stated the origin for all data sources. Additionally, we tuned hyperparameters using toy data and ran the real evaluation exactly once without intervention.
Randomization	For each task, we had to decide on which data to use for training and validation for training the networks, and we had to decide how to allocate the training and test data for evaluation. Wherever the original data publishers already made a decision we used their strategy. Otherwise, we randomly split the data using 2/3 of the data for training and 1/3 for testing. We made sure the same patient can only appear either in training or in the testing data for all evaluation tasks.
Blinding	The evaluation in our study did not include any subjective components. All data were based on objective findings, leaving no room for personal interpretation or bias. Consequently, there was no risk of bias that could have been mitigated by blinding.

Reporting for specific materials, systems and methods

We require information from authors about some types of materials, experimental systems and methods used in many studies. Here, indicate whether each material, system or method listed is relevant to your study. If you are not sure if a list item applies to your research, read the appropriate section before selecting a response.

Materials & experimental systems

n/a	Involved in the study
☒	☐ Antibodies
☒	☐ Eukaryotic cell lines
☒	☐ Palaeontology and archaeology
☒	☐ Animals and other organisms
☒	☐ Clinical data
☒	☐ Dual use research of concern
☒	☐ Plants

Methods

n/a	Involved in the study
☒	☐ ChIP-seq
☒	☐ Flow cytometry
☒	☐ MRI-based neuroimaging

Plants

Seed stocks	Not applicable
Novel plant genotypes	Not applicable
Authentication	Not applicable
