# Supplementary material for: Overcoming data scarcity in biomedical imaging with a foundational multi-task model
Source: Nat Comput Sci. 2024 Jul 19;4(7):495–509. doi: 10.1038/s43588-024-00662-z (PMC11288886; doi:10.1038/s43588-024-00662-z)
Supplement: Supplementary file 4 — The source code for reproducing the study results. For the most recent version, see https://github.com/FraunhoferMEVIS/MedicalMultitaskModeling. For the corresponding model weights of this study, see https://zenodo.org/records/11383543. [file 43588_2024_662_MOESM4_ESM.zip › code/medmnist_version/mtl-torch/mtl_torch/resources/jsonform.jinja2]

{{ user\_html }}
